# Complete chloroplast genome sequencing and comparative analysis of threatened dragon trees *Dracaena serrulata* and *Dracaena cinnabari*

**DOI:** 10.1038/s41598-022-20304-6

**Published:** 2022-10-06

**Authors:** Waqar Ahmad, Sajjad Asaf, Arif Khan, Ahmed Al-Harrasi, Abdulraqeb Al-Okaishi, Abdul Latif Khan

**Affiliations:** 1 Natural and Medical Sciences Research Centre, University of Nizwa, 616 Nizwa, Oman; 2 Genomics Group, Faculty of Biosciences and Aquaculture, Nord University, 8049 Bodø, Norway; 3 Environmental Protection Agency, Socotra, Yemen; 4 Department of Engineering Technology, University of Houston, Sugar Land, TX 77479 USA

**Keywords:** Photosynthesis, Plant ecology, Plant genetics

## Abstract

*Dracaena* (Asparagaceae family) tree is famous for producing "dragon blood"—a bioactive red-colored resin. Despite its long history of use in traditional medicine, little knowledge exists on the genomic architecture, phylogenetic position, or evolution. Hence, in this study, we sequenced the whole chloroplast (cp) genomes of *D. serrulata* and *D. cinnabari* and performed comparative genomics of nine genomes of the genus Dracaena. The results showed that the genome sizes range from 155,055 (*D. elliptica*) to 155,449 (*D. cochinchinensis*). The cp genomes of *D. serrulata* and *D. cinnabari* encode 131 genes, each including 85 and 84 protein-coding genes, respectively. However, the *D. hokouensis* had the highest number of genes (133), with 85 protein coding genes. Similarly, about 80 and 82 repeats were identified in the cp genomes of *D. serrulata* and *D. cinnabari*, respectively, while the highest repeats (103) were detected in the cp genome of *D. terniflora*. The number of simple sequence repeats (SSRs) was 176 and 159 in *D. serrulata* and *D. cinnabari* cp genomes, respectively. Furthermore, the comparative analysis of complete cp genomes revealed high sequence similarity. However, some sequence divergences were observed in *accD*, *matK*, *rpl16*, *rpoC2*, and *ycf1* genes and some intergenic spacers. The phylogenomic analysis revealed that *D. serrulata* and *D. cinnabari* form a monophyletic clade, sister to the remaining *Dracaena* species sampled in this study, with high bootstrap values. In conclusion, this study provides valuable genetic information for studying the evolutionary relationships and population genetics of *Dracaena*, which is threatened in its conservation status.

## Introduction

Dracaena is an important genus from the family Asparagaceae that includes wild and indoor exquisite plants^1^. The genus comprises 190 species^2^ and is also known as Dragon trees. These are distributed across the drylands in Africa, Arabia, and the Americas^3^. In response to incisions, these plants produce a red resin called “Dragon Blood” that is medicinally important and has an ancient history in traditional herbal medicine^4^. The resin has been known to act as an anti-cancer, hemostatic, anti-ulcer, anti-viral, anti-microbial, anti-inflammatory, and anti-oxidant^5^. Dracaena resin is also used for giving colors to certain materials like toothpaste, varnishes, and plasters^6^. The highest levels of species diversity occur in tropical Africa and Southeast Asia. These species grow in various habitats, including tropical monsoon, semi-evergreen, and evergreen rain forests. Some species grow in specialized habitats such as escarpments, littoral forest edges, and riverbeds with strongly fluctuating water levels, where they become facultative rheophytes^7^.

**Figure 1:**
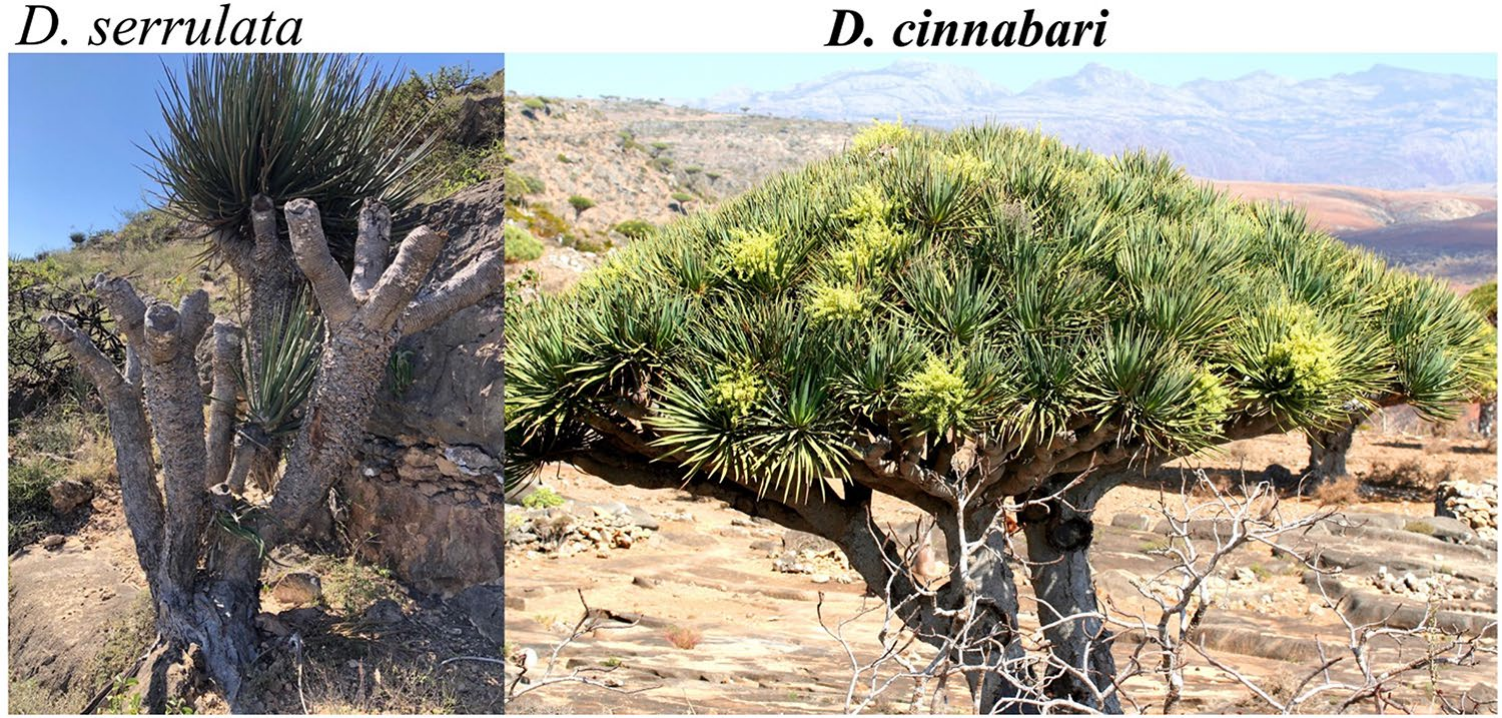
The dragon tree plants and their habitat. *D. serrulata* (A) and *D. cinnabari* (B).

Among *Dracaena* species, *D. serrulata* and *D. cinnabari* (Fig. 1) are regional, endemic species found in southern Oman, Saudi Arabia, and Yemen (Socotra Island). These endangered species are currently threatened by mining operations, agriculture, drought, and possibly climate change. The known populations are threatened by grazing (camels, goats, and sheep) during the dry season^8–10^. *Dracaena,* along with other globally important genera *Sansevieria* Thunb and *Pleomele* Salisb (family Asparagaceae and Nolinoideae subfamily) are collectively referred to as ‘dracaenoids’. These have had various taxonomic and evolutionary unsolved problems since the eighteenth century^11,12^. The classification of these three genera was always unclear, and that's why these were shifted from one family to another like Agavaceae, Liliaceae^13,14^, Dracaenaceae^7,15,16^, Ruscaceae^11^, and lately in Asparagaceae^17^ over a period of times. Due to similar floral characters, *Sansevieria* and *Dracaena* were believed to be synonymous. However, their stature, leaf morphology, and plant habitats have distinct variations. Similarly, the dracaenoid genera have ambiguous systematic relationships, and extensive evolutionary history and biogeographic studies are needed^18^.

The ambiguity is associated with *Dracaena* species identification and intra-generic relationships, including sub-species of *D. serrulata*^2^. According to Marrero et al.^3^ assessment based on morphological and ecological characteristics, the *D. draco* (Macaronesian species) would show closer affinities with the *D. cinnabari* (Socotran species) and *D. tamaranae*, which is located in the Horn of Africa, is more closely related to the *D. serrulata* (Arabian species). However, Durán et al.^19^ reported that based on barcoding genes (*rbcL* and *matK*) and intergenic spacers (*trnQ-rps16* and *rpl32-trnL*), *D. cinnabari* (Socoteran) is more closely related to *D. serrrulata* (Omani species) than *D. draco* (Macaronesian). Genomic studies can resolve such species-relatedness, which is minimal for the genus *Dracaena*^20^. Next-generation sequencing combined with bioinformatic analysis can also help solve key taxonomic and genetic diversity issues^21^.

In this case, the chloroplast is one of the most important organelles and has its own independently replicating genome called chloroplast genome or plastome^22^. Chloroplast genome possesses a typical quadripartite structure having a large single-copy region (LSC), small single-copy region (SSC) and a pair of inverted repeats (IRa and IRb), which are mirror images of each other^23^. The chloroplast genome is highly conserved among typical land plants compared to other genomes present in the plant cell like mitochondria and nuclear genome^24^. Despite the conservative nature of the chloroplast genome, it still has variations like insertion and deletion and single nucleotide polymorphism, which provides sufficient information in plant identification^25,26^. Many chloroplast derived markers are used in the plant phylogenetic, population genetics, and phylogeographic analyses due to their low recombination, low nucleotide substitutions rate, and uniparental inheritance^27^. Many chloroplast genomes such as *trnH-psbA*, *rbcL*, and *matK* were commonly used as DNA barcodes for plant identification and discrimination of sub-species^17^.

In some cases, these barcodes cannot differentiate especially between the closely related species within Dracaenoid genera^28^, due to the little variation in the loci^27,29^. Complete chloroplast genome sequencing coupled with comparative analysis allows advanced phylogenetic reconstruction and can be used as super-barcodes to resolve identification at lower taxonomic levels^27,30^. Looking at these challenges, in the current study, we aim to sequence the *D. serrulata* and *D. cinnabari* and perform comparative chloroplast genome analysis to explain the basis of genome architecture and divergences across *Dracaena* species. Hence, we report the complete chloroplast genomes sequences of *D. serrulata* and *D. cinnabari*. Both the species and other species in the genus possess the least genomic information. Hence, current datasets will help understand the genome architecture, comparative genomics with related species, and in-depth phylogeny of *Dracaena* species.

## Methodology

### Sample collection

Fresh young leaves were collected from the *D. serrulata* and *D. cinnabari* plants growing in the Dhofar region (wild) of Oman. The habitat climate is arid with a low precipitation rate and temperate (25–46 °C). All plant specimens used for this study were collected from the wild to the best of our knowledge in compliance with local, institutional, national, or international regulations at the time of collection. A permission letter was retrieved from the Director-General of Nature Conservation, Ministry of Environment and Climate Affairs, Sultanate of Oman. The fresh specimens of *D. cinnabari* were donated by the Environmental Protection Authority Socotra, Yemen. The voucher specimen numbered UoN-DS1 (*D. serrulata*) and UoN-DC1 (*D. cinnabari*) were deposited in the University of Nizwa herbarium center. The identification of plants was carried out by Saif Al-Hathmi, an expert taxonomist at the Oman Botanical Garden in Muscat, Oman. The collected materials were transported in liquid nitrogen or dry ice and stored at − 80 °C for further processing.

### DNA extraction and sequencing

With brief modifications, the cp DNA was isolated from collected samples as described in Shi et al.^31^. The construction of genomic libraries was carried out as per provided instructions (Life Technologies USA, Eugene, OR, USA). To arrange the cp DNA into 400 bp fragments (enzymatically) for libraries, the Ion Shear™ Plus Reagents kit and Ion Xpress™ Plus gDNA Fragment Library kit were used. Qubit 3.0 fluorometer and bioanalyzer (Agilent 2100 Bioanalyzer system, Life Technologies USA) were used to quantify the prepared libraries. The amplification of the template was performed using Ion OneTouch™ 2. The Ion OneTouchTM ES enrichment system enriched the amplified templates using Ion 530 and 520 OT2 reagents. Ion S5 protocol of sequencing was followed for loading the sample on S5 530 chip.

### Genome assembly and annotation

The number of raw reads obtained for *D. serrulata* and *D. cinnabari* were 14,654,144 and 16,888,126, respectively. The reads were first screened for a Phred score < 30 to remove low-quality sequences. To ensure the accuracy of cp genome assembly, we employed two methods to assemble the cp genome. In the first method, obtained reads of cp genomes *D. serrulata* and *D. cinnabari* were mapped to the reference genome of *D. cochinchinensis* (MF943127) and *D. combodiana* (MN20094), respectively, by Geneious Pro (v.10.2.3) software using Bowtie2 (v.2.2.3)^32,33^. Assembly means coverage of *D. serrulata* was 876X, and *D. cinnabari* was 768X. In the second method, the cp genome of *D. serrulata* and *D. cinnabari* were de novo assembled using the GetOrganelle pipeline^34^, with SPAdes 3.10.1 assembler^35^. The cp genomes *D. serrulata* and *D. cinnabari* were annotated using CpGAVAS and DOGMA (http://dogma.ccbb.utexas.edu/, China)^36^. The tRNAs can-SE detected the tRNA genes (v.1.21)^37^. Intron boundaries, manual alteration and start and stop codon adjustments of genomes were carried out using Geneious Pro (v.10.2.3)^33^ and tRNAs can-SE^37^ by comparing the cp genomes to reference genomes. OGDRAW^38^ was utilized to illustrate the structural features in cp genomes.

### Repeat identification

The determination of palindromic, forward and reverse repeats was performed using the online tool REPuter^39^ with 8 bp minimum repeat size and 50 maximum computed repeats. Furthermore, MISA software^40^ with conditions of ≥ 10 repeat units for 1 bp repeats; ≥ 8 repeat units for 2 bp repeats; ≥ 4 repeat units for 3 and 4 bp repeats and ≥ 3 repeat units for 5 and 6 bp repeats was used to calculate Simple sequence repeats (SSRs) and tandem repeats were calculated by Tandem Repeats Finder v.4.09^41^.

### Genome divergence

The sequence divergence in shared genes and complete cp genomes of *D. serrulata* and *D. cinnabari,* and other closely related species were determined. Multiple sequence alignment was performed via comparative analysis, and the gene order was compared to clarify the missing and ambiguous gene annotation. The cp genomes were aligned with default parameters using MAFFT version 7.222^42^ with default parameters. Kimura’s two parameter model (K2P)^43^ was utilized to find the pairwise sequence divergence. The relative synonymous codon usage (RSCU) value analysis and variable sites (Pi) were calculated through sliding window analysis using DnaSP software version 6.13.03^44^. The mVISTA^45^ in shuffle-LAGAN mode was used to determine the genomic divergence while using cp genome of *D. serrulata* as a reference.

### Phylogenetic analysis

To resolve the phylogenetic position of *D. serrulata* and *D. cinnabari* within the subfamily of Nolinoideae a total of 44 cp genomes were retrieved from NCBI database. Four *Asparagus* species, *A. schoberioides*, *A. officinalis*, *A. racemosus* and *A. setaceus* were selected as outgroups. The first tier alignment of complete cp genomes was performed according to the cp genome structure and conserved gene order^46^. The phylogenetic trees were constructed using four methods by employing the setting described previously by Asaf et al.^48^. Neighbour-joining (NJ) and maximum likelihood (ML) were implemented in MEGA 6^49^; Bayesian inference (BI) was employed in MrBayes 3.1.2^50^; and maximum parsimony (MP) by using PAUP version 4.0^51^. For the ML run, the parameters were optimized by BIONJ tree^52^ as the starting tree with 1000 bootstrap replicates by employing the Kimura 2-parameter model with invariant sites gamma-distributed rate heterogeneity. For Bayesian inference, the best substitution model GTR + G was tested by jModelTest version v2.1.02100 according to the Akaike information criterion (AIC) for Bayesian posterior probabilities (PP) in BI analyses. The Markov Chain Monte Carlo (MCMC) method was run using four incrementally heated chains across 1,000,000 generations, starting from random trees and sampling 1 out of every 100 generations. To estimate the posterior probabilities, the values of first 30% of trees were discarded as burn-in. Similarly, the maximum parsimony run was based on a heuristic search with 1000 random addition of sequence replicates with the tree-bisection-reconnection (TBR) branch-swapping tree search criterion. In the second tier, 66 shared protein-coding genes from 46 cp genomes from subfamily Nolinoideae were aligned using MAFFT version 7.22294 under default parameters and making various manual adjustments to preserve and improve reading frames in the second tiers of phylogenetic analysis. The above four aforementioned phylogenetic inference models (ML, NJ, BI and MP) were employed to construct trees using 66 concatenated genes as mentioned above and suggested by Asaf et al.^53^.

## Results and discussion

**Figure 2:**
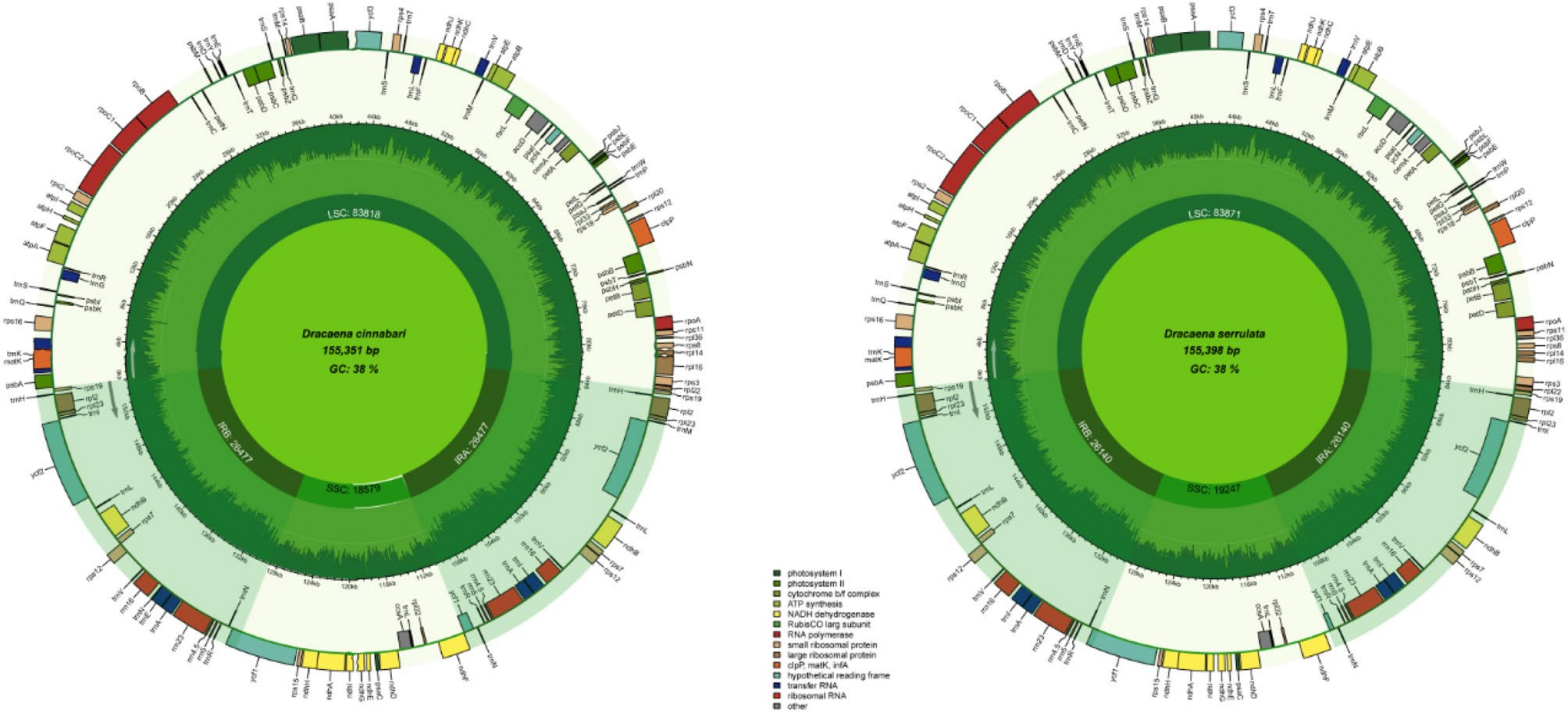
Genome Map of the *D. serrulata* and *D. cinnabari* cp genomes. Thick lines represent inverted repeat regions (IRs). IRs split the cp genome into large single copies (LSC) and single small copies (SSC) regions. The counter-clockwise transcribing genes are drawn outside while the clockwise are drawn inside the circle. Genes related to different functional groups are color coded. The GC and AC content is represented by the circle's dark and light green shades.

The results showed that the cp genomes of *D. serrulata* (MT408026) and *D. cinnabari* (OK235335) have the typical quadripartite structures like other related plants^54–56^ with a genome size of 155,398 bp and 155,351 bp respectively. Both the cp genomes comprised of 4 distinctive parts in which the LSC (83,871 bp, 83,818 bp) and SSC (19,247 bp, 18,579 bp) are separated by two IRs (26,140 bp, 26,477 bp) (Fig. 2, Table 1). The cp genomes of *D. serrulata* and *D. cinnabari* were analyzed and compared with *D. angustifolia*, *D. cambodiana*, *D. cochinchinensis*, *D. cochinchinensis2*, *D. draco*,*D. elliptica*, *D. fragrans*, *D. hokouensis* and *D. terniflora* (Table 1), which are closely related and belongs to the same genus. The sizes of these cp genomes range from 155,055 bp (*D. elliptica*) to 155,449 bp (*D. cochinchinensis*), as shown in Table 1. The cp genomes of *D. serrulata* and *D. cinnabari* encodes a total of 131 genes like all compared cp genomes except *D. cambodiana*, *D. cochinchinensis* and *D. elliptica,* which encode 130 genes while *D. hokouensis* encodes 133 genes. Similarly, among the total genes encoded by cp genomes of *D. serrulata* and *D. cinnabari,* 85 and 84 are protein-coding genes, respectively (Table 1). Furthermore, D. serrulata and D. cinnabari's cp genomes encode eight rRNA and 38 tRNA genes, respectively (Fig. 2). Similar results were reported previously in other angiosperms^57–59^. Among the protein-coding genes 12 genes (*rps11,12, 14, 15, 16, 18, 2, 3, 4, 7, 7, 8*) code for small ribosomal subunits, 9 genes (*rpl14, 16, 2, 0, 22, 23, 32, 33, 36*) for large ribosomal subunits, 44 genes (Table 2) photosynthesis related proteins, 4 (*rpoA, rpoB, rpoC1, rpoC2*) DNA dependent RNA polymerase, and 8 genes (*accD, ccsA, cemA, matK, ycf1, ycf2, ycf3, ycf4*) code for other proteins (Table 2). Furthermore, 20 genes containing introns were identified in 18 genes containing a single intron whereas two genes (*ycf3*, *clpP*) had two introns and three exons (Table 3). The *trnK*-UUU gene was identified with the largest intron (2,568 bp) and *rpl2* gene with the smallest intron (652 bp). The *rps12* gene was trans-spliced; the 5′ end exon was detected in the LSC region and the 3′ exon was identified in the IR region, as in most other angiosperms. These results are consistent with previous reports^60–62^. The overall GC content of *D. serrulata* and *D. cinnabari* cp genomes was 37.6% and 37.5%, respectively, similarly found in other cp genomes (Table 1) as reported previously^28^.

**Table 1:** Chloroplast genomes features summary of *D. serrulata, D. cinnabari* and related species of *Dracaena* genus.

	*D. serrulata*	*D. cinnabari*	*D. angustifolia*	*D. cambodiana*	*D. cochinchinensis*	*D. cochinchinensis2*	*D. draco*	*D. elliptica*	*D. fragrans*	*D. hokouensis*	*D. terniflora*
Size (bp)	155,398	155,351	155,332	155,291	155,449	155,182	155,422	155,055	155,340	155,183	155,347
Overall GC contents	37.6	37.5	37.5	37.6	37.6	37.5	37.6	37.5	37.5	37.5	37.5
LSC size in bp	83,871	83,818	83,803	83,752	83,907	83,702	83,942	83,621	83,976	83,703	83,794
SSC size in bp	19,247	18,579	18,465	18,489	18,492	18,466	18,472	18,456	18,494	18,466	18,493
IR size in bp	26,140	26,477	26,530	26,525	26,525	26,507	26,504	26,489	26,525	26,507	26,530
Protein coding regions size in bp	78,777	77,658	78,732	77,202	77,187	78,708	78,537	77,130	78,744	78,297	78,744
tRNA size in bp	3061	2936	2873	2874	2874	2866	2867	2874	2874	2867	2873
rRNA size in bp	9050	9040	9050	9050	9050	9050	9050	9050	9050	9050	9050
Number of genes	131	131	131	130	130	131	131	130	131	133	131
Number of protein coding genes	85	84	85	84	84	85	85	84	85	85	85
Number of rRNA	8	8	8	8	8	8	8	8	8	8	8
Number of tRNA	38	38	38	38	38	38	38	38	38	38	38
Genes with introns	22	22	23	23	23	23	23	23	23	23	23
Gene Bank Accession Number	MT408026	OK235335	MN200193	MN200194	MF943127	MN200195	MN990038	MN200196	MW123093	MN200197	MN200198

**Table 2:** Gene composition in *Dracaena species* cp genomes.

Category of genes		Group of genes
Genes for photosynthesis	Subunits of ATP synthase	atpA, atpB, atpE, atpF, atpH, atpI
Genes for photosynthesis	Subunits of photosystem II	psbA, psbB, psbC, psbD, psbE, psbF, psbI, psbJ, psbK, psbL, psbM, psbN, psbT, psbZ
Genes for photosynthesis	Subunits of NADH-dehydrogenase	ndhA, ndhB, ndhB, ndhC, ndhD, ndhE, ndhF, ndhG, ndhH, ndhI, ndhJ, ndhK
Genes for photosynthesis	Subunits of cytochrome b/f complex	petA, petB, petD, petG, petL, petN
Genes for photosynthesis	Subunits of photosystem I	psaA, psaB, psaC, psaI, psaJ
Genes for photosynthesis	Subunit of rubisco	rbcL
Self-replication	Large subunit of ribosome	rpl14, rpl16, rpl2, rpl2, rpl20, rpl22, rpl23, rpl23, rpl32, rpl33, rpl36
Self-replication	DNA dependent RNA polymerase	rpoA, rpoB, rpoB, rpoB, rpoC1, rpoC2
Self-replication	Small subunit of ribosome	rps11, rps12, rps12, rps14, rps15, rps16, rps18, rps2, rps3, rps4, rps7, rps7, rps8
Other genes	Subunit of Acetyl-CoA-carboxylase	accD
Other genes	c-type cytochrom synthesis gene	ccsA
Other genes	Envelop membrane protein	cemA
Other genes	Maturase	matK
Unkown	Conserved open reading frames	ycf1, ycf2, ycf3, ycf4

**Table 3:** Introns and exons lengths for the splitting genes in cp genomes of *D. serrulata* and *D. cinnabari.*

Gene	Start		End		ExonI (bp)		IntronI (bp)		ExonII (bp)		IntronII (bp)		ExonIII (bp)	
	DS	DC	DS	DC	DS	DC	DS	DC	DS	DC	DS	DC	DS	DC
trnK-UUU	1513	1513	4157	4157	37	37	2568	2568	40	40				
rps16	4789	4789	5910	5910	46	46	867	867	209	209				
trnG-GCC	9131	9131	9906	9906	23	23	716	716	37	37				
atpF	11,854	11,854	13,230	13,230	145	145	828	828	404	404				
rpoC1	20,640	20,640	23,415	23,415	432	432	718	718	1626	1626				
ycf3	42,150	42,150	44,126	44,126	126	126	731	731	220	220	739	739	161	161
trnL-UAA	46,962	46,962	47,593	47,593	35	35	547	547	50	50				
trnV-UAC	52,093	52,093	52,754	52,754	39	39	586	586	37	37				
clpP	70,044	70,497	72,097	72,016	69	69	825	819	291	291	644	621	225	225
petB	74,979	74,979	76,381	76,381	7	7	752	752	644	644				
petD	76,586	76,586	77,830	77,830	8	8	732	732	505	505				
rpl2	84,455	84,455	85,928	85,928	391	391	652	652	431	431				
ndhB	94,954	94,954	97,185	97,185	775	775	699	699	758	758				
trnA-UGC	103,803	103,803	104,690	104,690	38	38	815	815	35	35				
ndhA	120,271	120,271	122,444	122,444	559	559	1076	1076	539	539				
trnA-UGC	134,580	134,580	135,467	135,467	38	38	815	815	35	35				
ndhB	142,085	142,085	144,316	144,316	775	775	699	699	758	758				
rps12														
trnG-GCC	9131	9035	9906	9811	23	23	716	706	37	48				
trnI-GAU	135,532	102,671	136,545	103,689	42	32	937	947	35	40				

### Repeats and simple sequence repeats SSR analysis in Cp genomes

**Figure 3:**
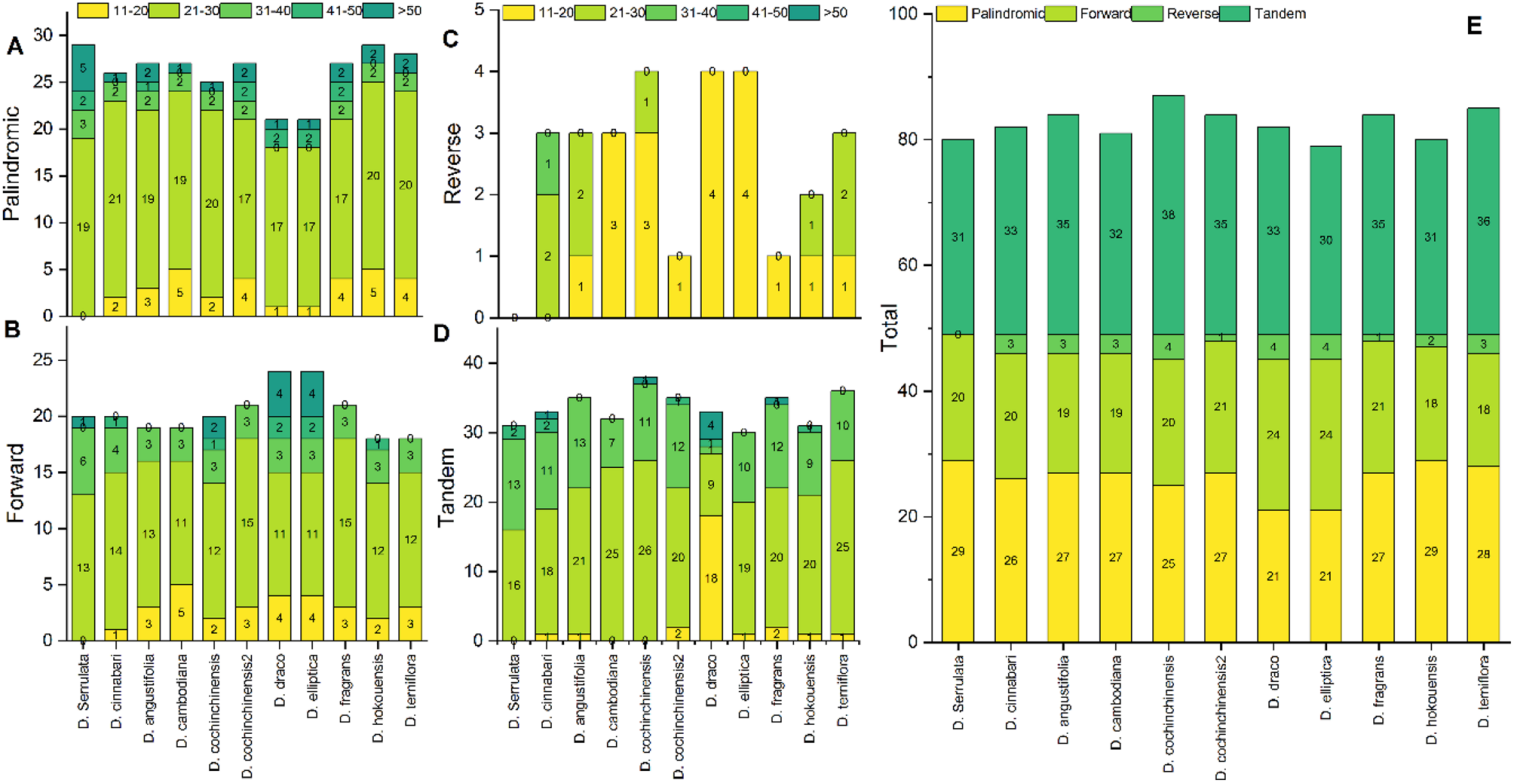
Repetitive sequences in *D. serrulata, D. cinnabari* and related *Dracaena* species cp genomes. (**A**) A total number of repetitive sequences in cp genomes, (**B**) Lengthwise frequency of palindromic repeats (**C**) Lengthwise frequency of forward repeats (**D**) Lengthwise frequency of reverse repeats (**E**) Lengthwise frequency of tandem repeats.

A total of 80 and 82 repeats were identified in *D. serrulata* and *D. cinnabari,* respectively. In contrast, the cp genome of *D. terniflora* had the highest number of total repeats (103) and *D. elliptica* had the minimum (79). In *D. serrulata* and *D. cinnabari,* the palindromic repeats were 29 and 26, respectively (Fig. 3A). Similarly, both sequenced cp genomes had the forward repeats of 20 each (Fig. 3B) whereas the reverse repeats identified were zero in *D. serrulata* and 3 in *D. cinnabari* (Fig. 3C). Furthermore, the tandem repeats were also identified for both sequenced genomes, 31 and 33, respectively (Fig. 3D). Although, the highest and lowest number of forward repeats were detected in cp genome of *D. terniflora* (36) and *D. hokouensis* (19), while the reverse repeats were highest in *D. cochinchinensis, D. draco* and *D. elliptica* (4) and zero in *D. serrulata.* Most palindromic repeats were detected in *D. serrulata* and *D. hokouensis* i.e. 29. Similarly, the tandem repeats were most in the cp genome of *D. cochinchinensis* (38) and least in *D. elliptica* (30). The total number of repeats was highest (87) in *D. cochinchinensis* (Fig. 3E).

**Figure 4:**
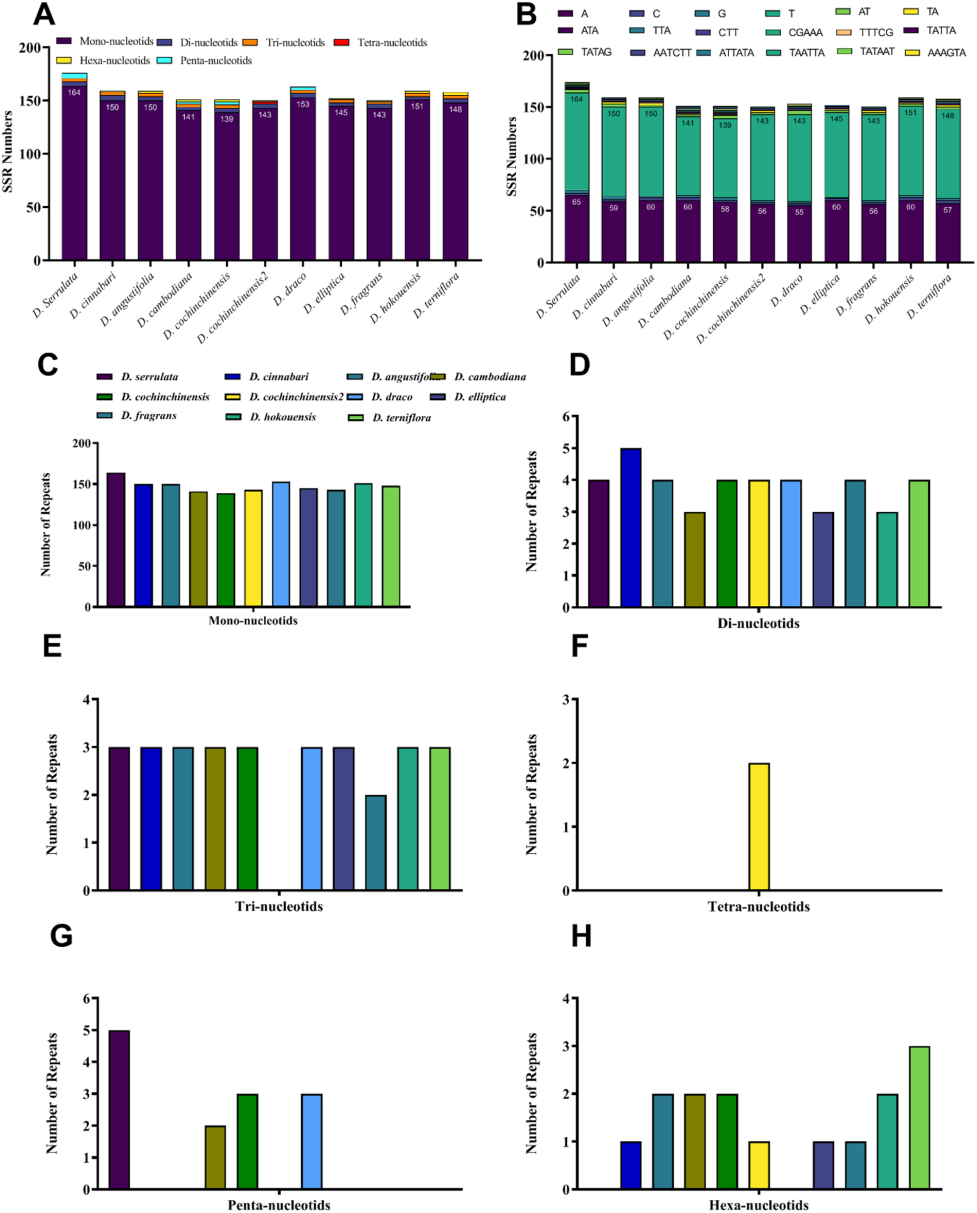
Simple sequence repeats (SSRs) in *D. serrulata, D. cinnabari,* and *related Dracaena* species cp genomes. (**A**) Total number of SSRs in cp genomes, (**B**) SSR motif frequency in cp genomes, (**C**) Mono-nucleotides SSRs (**D**) Di-nucleotides SSRs, (**E**) Tri-nucleotides SSRs, (**F**) Tetra-nucleotides SSRs, (**G**) Penta-nucleotides SSRs and (**H**) Hexa-nucleotides SSRs.

Simple sequence repeats (SSR) are used as genetic markers in evolutionary and population genetics studies^63^. These repeats also known as microsatellites are usually comprised of 1–6 bp repeat units^64^. Furthermore, SSRs are important because their relative lack of recombination, maternal inheritance, and haploid nature make them potential candidates for phylogenetic studies. SSRs play a role in estimating genetic variation, gene flow analysis, and studying the population history in plants and animals^65,66^. In this study, we analyzed SSRs in the cp genomes of *D. serrulata and D. cinnabari* and nine other *Dracaena* species Fig. 4A and B)*.* Interestingly, the highest number of SSRs were identified in *D. serrulata* (176) followed by *D. draco* (163). In *D. cinnabari, D. angustifolia* and *D. hokouensis,* the identified SSRs were 159. The least number of SSRs were identified in *D. cambodiana* and *D. cochinchinensis,* which were 152 (Fig. 4A). Mononucleotide repeats were the most detected SSRs (Fig. 4C). The highest number of mono-nucleotide SSRs were detected in *D. Serrulata* (164), followed by *D. hokouensis* (151). The highest number of di-nucleotide SSRs were detected in the sequenced cp genome of *D. cinnabari* (5), followed *D. serrulata* (4) (Fig. 4D), while the tri-nucleotide SSRs were 3 in cp genomes of *D. serrulata* and *D. cinnabari* along with other compared cp genomes except in *D. fragrance* which were two and *D. cochinchinensis2* with no tri-nucleotides (Fig. 4E). A total of 2 tetra-nucleotide SSRs were detected only in the cp genome of *D. cochinchinensis*. In contrast, in this study, the remaining cp genomes had no tetra-nucleotide SSRs, including the sequenced cp genomes of *D. serrulata* and *D. cinnabari* (Fig. 4F). The penta-nucleotide SSRs detected in *D. serrulata* were 5, while the *D. cinnabari* had zero (Fig. 4G). Contrastingly the hexanucleotide SSRs was found in only the *D. cinnabari* cp genome, as shown in Fig. 4H. Likewise, patterns in *Dracaena* and other angiosperms cp genomes were also reported previously^67,68^. Our results agree with the recent studies reporting that identified SSRs in cp genomes are made of polyadenine or polythymine repeats. The contrary is with guanine (G) and cytosine (C). Therefore, the cp genomes of *D. serrulata* and *D. cinnabari* are rich in ‘AT’ content, as reported previously^69–71^. As per earlier reports, the SSRs are randomly distributed across the cp genomes, revealing important information for selecting molecular markers for polymorphism (inter and intra-specific)^72,73^. The current results are in synergy with previous reports of angiosperms indicating the dominating abundance of ‘A’ or ‘T’ mono-nucleotides SSRs in cp genomes and resulting in ‘AT’ rich cp genomes^74,75^.

### Comparative analysis and sequence divergence analyses

**Figure 5:**
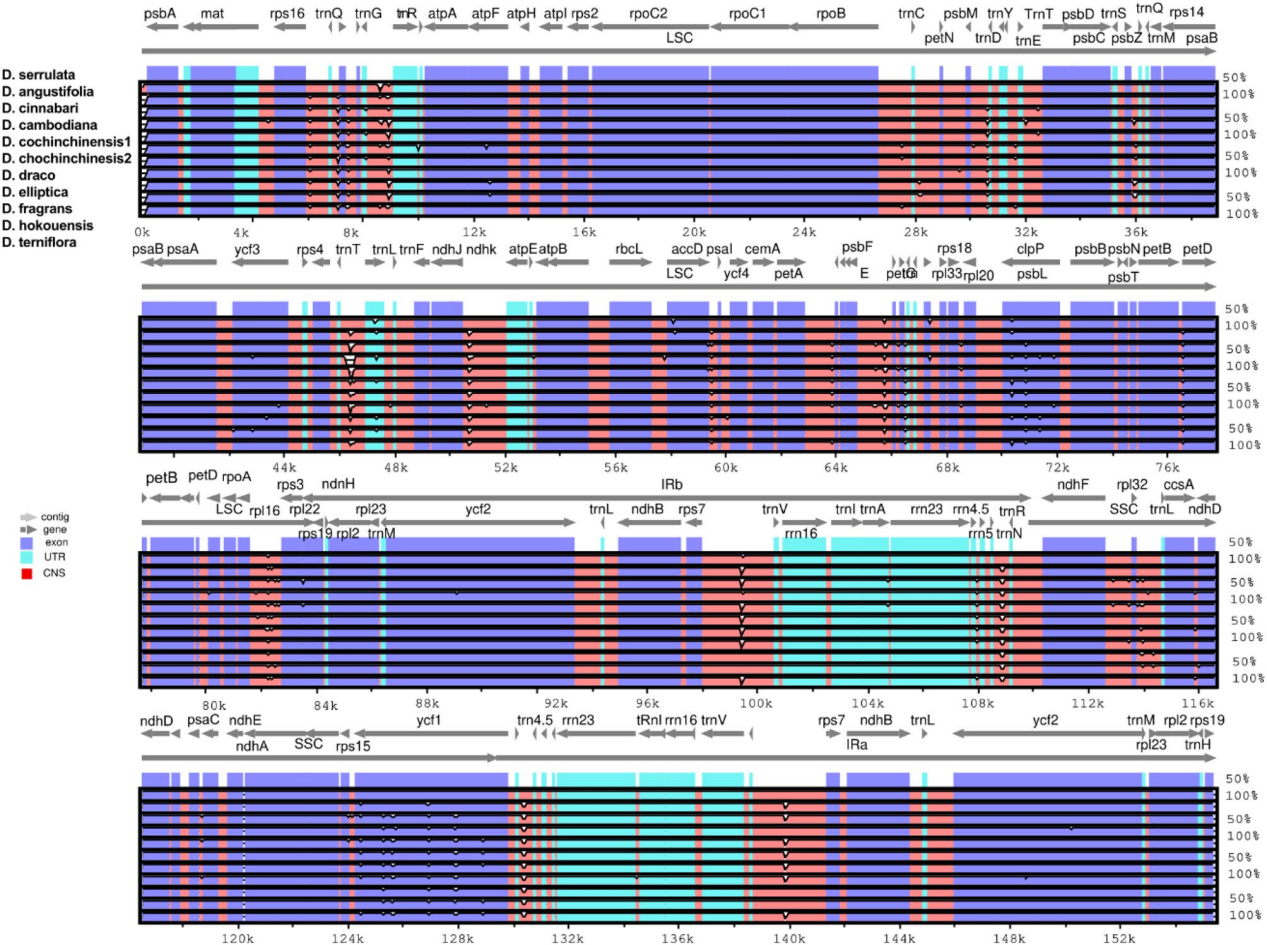
Visual alignment of *D. serrulata, D. cinnabari,* and related *Dracaena* species cp genomes. VISTA-based identity plot showing sequence identities among eleven *Dracaena* species, using *D. serrulata* as a reference. Genome regions are color-coded as protein-coding, rRNA coding, tRNA coding, or conserved non-coding sequences (CNS). The x-axis represents the coordinate in the chloroplast genome. Annotated genes are displayed along the top. The sequences similarity of the aligned regions is shown as horizontal bars indicating the average percent identity between 50 and 100%.

Comparative analysis of the cp genome plays a pivotal role in understanding plant species' genetic diversity and evolutionary relationships^22,76^ The cp genomes *D. serrulata* and *D. cinnabari* were compared to the closely related species for sequence divergence. The cp genome of *D. serrulata* was selected as a reference genome. The cp genomes of *D. serrulata* and *D. cinnabari* along with all the compared cp genomes, were highly conserved. All aligned sequences exhibit high similarities with only a few regions sequence variations in non-coding regions (Fig. 5). Interestingly, a higher degree of divergence was observed in non-coding regions in all cp genomes compared to the coding areas reported previously^77,78^. The current results revealed the high similarity of cp genome sequences of all species included in the study, suggesting that the cp genomes of *Dracaena* genus are highly conserved as reported for *Dracaena*^28^ and *Camellia* genus^79^. The *petD, and clpP* genes in the LSC region, and the *ycf1* gene in the SSC region showed sequence divergence in the coding areas across all compared species, and these results agree with^21,28,71,80^.

Moreover, in IR regions, the *rps19* gene showed sequence divergence in the cp genomes of *D. cinnabari* and *D. cochinchinensis. In contrast,* the *ycf2* gene showed variation in the cp genome of *D. cambodiana.* In the LSC region, *accD atpF*, *ycf3,* and *rps15* genes showed sequence divergence in some cp genomes compared to the *D. serrulata* cp genome (Fig. 5). Furthermore, in the non-coding areas such as *rsp16-trnT*, *rps4-trnL*, and *petE-trnG* in LSC while *rps7-trnV* in SSC showed sequence divergence across all the compared cp genomes, likewise pattern of divergence was also reported previously^78,79,81^.

**Figure 6:**
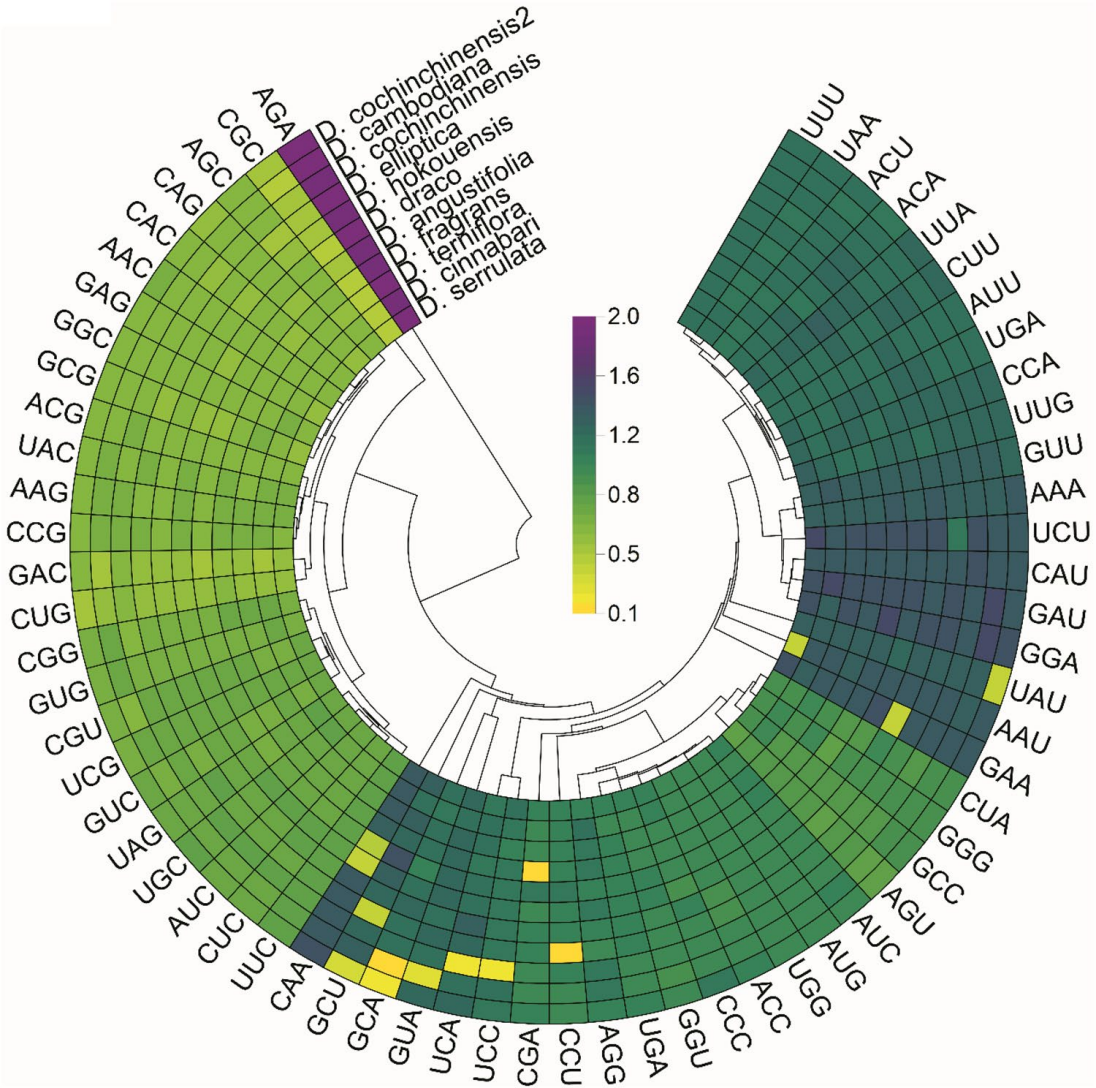
Heatmap plot of codon distribution of all shared protein-coding genes in 11 *Dracaena* species. Color key: yellow indicates lower, green indicates moderate, while purple indicates higher RSCU values.

Moreover, the average pairwise sequence divergence among the complete cp genomes (Table S2) and shared genes (Table S3) was calculated. *D. cinnabari* cp genome showed an average pairwise sequence divergence of 0.003. The cp genome of *D. cinnabari* showed the highest average pairwise sequence divergence with *D. cochinchinensis* and *D. fragrance* (0.0077). Other cp genomes included in the study and previous reports also showed similar results^48,71^. The most divergent genes were *accD, matK, rpl16, rpoC2,* and *ycf1*. The highest pairwise sequence divergence was identified for *rpl16* (0.03) (Table S3). Similar results are also reported by Zhang, et al.^28^. Similarly, the relative synonymous codon usage (RSCU) value analysis was performed using coding regions of 10 *Dracaena* cp genomes. The most abundantly used codons were A/U-ending codons. These results exhibited a higher codon usage toward A/U- endings than G/C-ended codons in all cp genomes of *Dracaena* species^28,82,83^. Codons like CAA, GCU, GCA, and GUA UAC (yellow colored) have less than one RSCU value in one or more cp genomes (Fig. 6). Whereas the highest RSCU value was recorded for AGA (2) across all cp genomes, similar results were reported for *Punica granatum*^84^ and *D. draco*^28^. The codon characteristic pattern and frequency of usage are given in Table S1. The most frequently used codon was AAA (*n* = 2,036, 51.5%) in these genomes, which encodes lysine amino acid. In contrast, the least used codon was GCG (*n* = 257, 5.2%), coding the arginine amino acid (Table S1); these results agree with earlier reports^28,85^.

**Figure 7:**
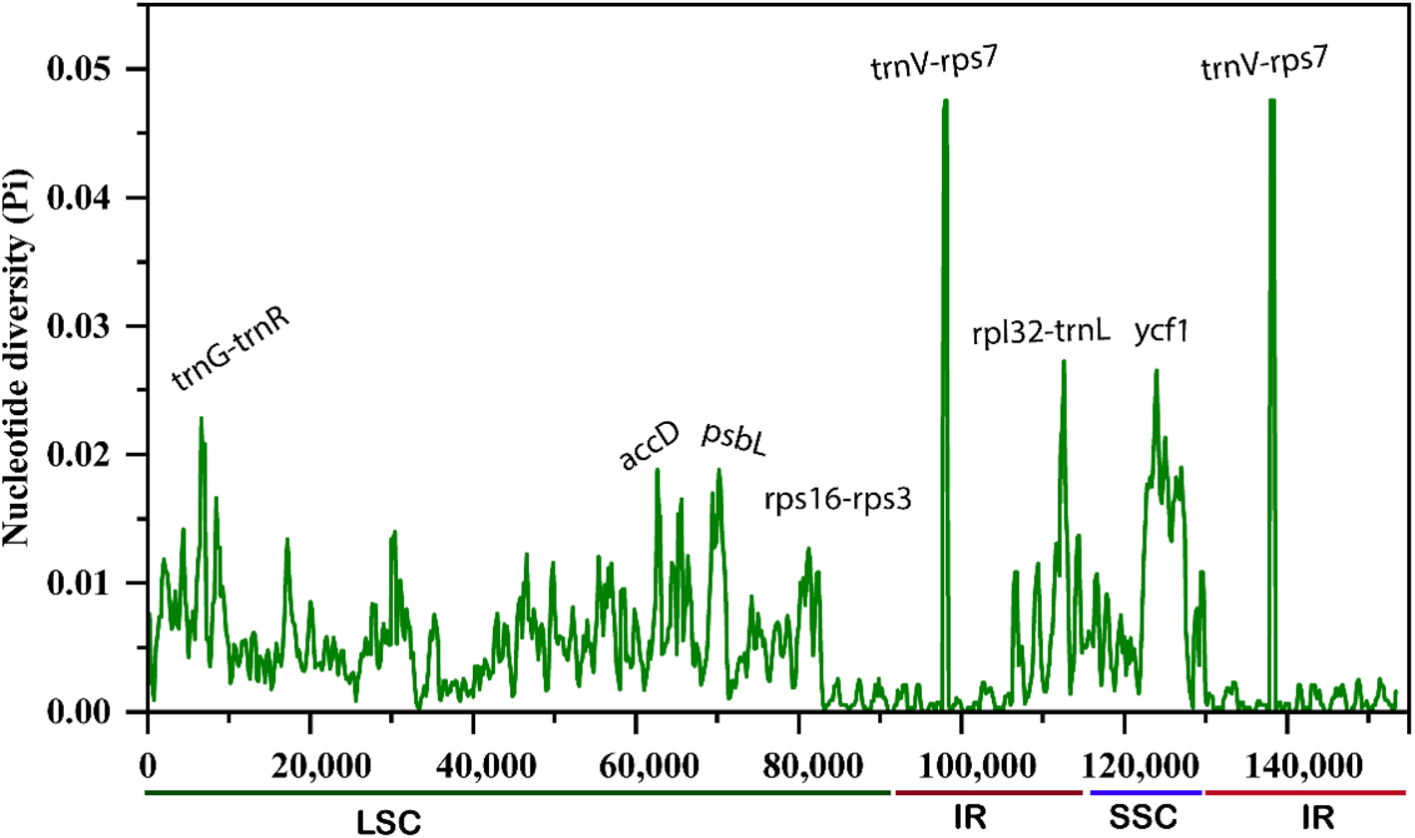
Sliding window analysis of nucleotide variability among the *Dracaena* species cp genomes (window length: 600 bp; step size: 200 bp).

Similarly, the nucleotide diversity (Pi) values were calculated in these cp genomes (Fig. 7). The Pi values ranged from 0 to 0.024 (LSC), 0 to 0.027 (LSC), and 0 to 0.049 (IRs) with a mean of 0.0030, which indicates that the variation is slight among these cp genomes and are highly conserved, similar variation patterns were previously reported in angiosperm cp genomes^86^. Furthermore, the IR region showed higher Pi values than LSC and SSC reported^87^. However, some genes like *accD*, *psbL* (LSC), and *ycf1* (SSC) showed higher Pi values of 0.02, 0.02, and 0.026 than other protein-coding genes. Similarly, the *trnV-rps7* (IR region) showed the highest Pi value of 0.05. these results also agree with mVISTA divergence analysis and previous reports^21,88,89^.

### Contraction and expansion of IRs and single copy regions

**Figure 8:**
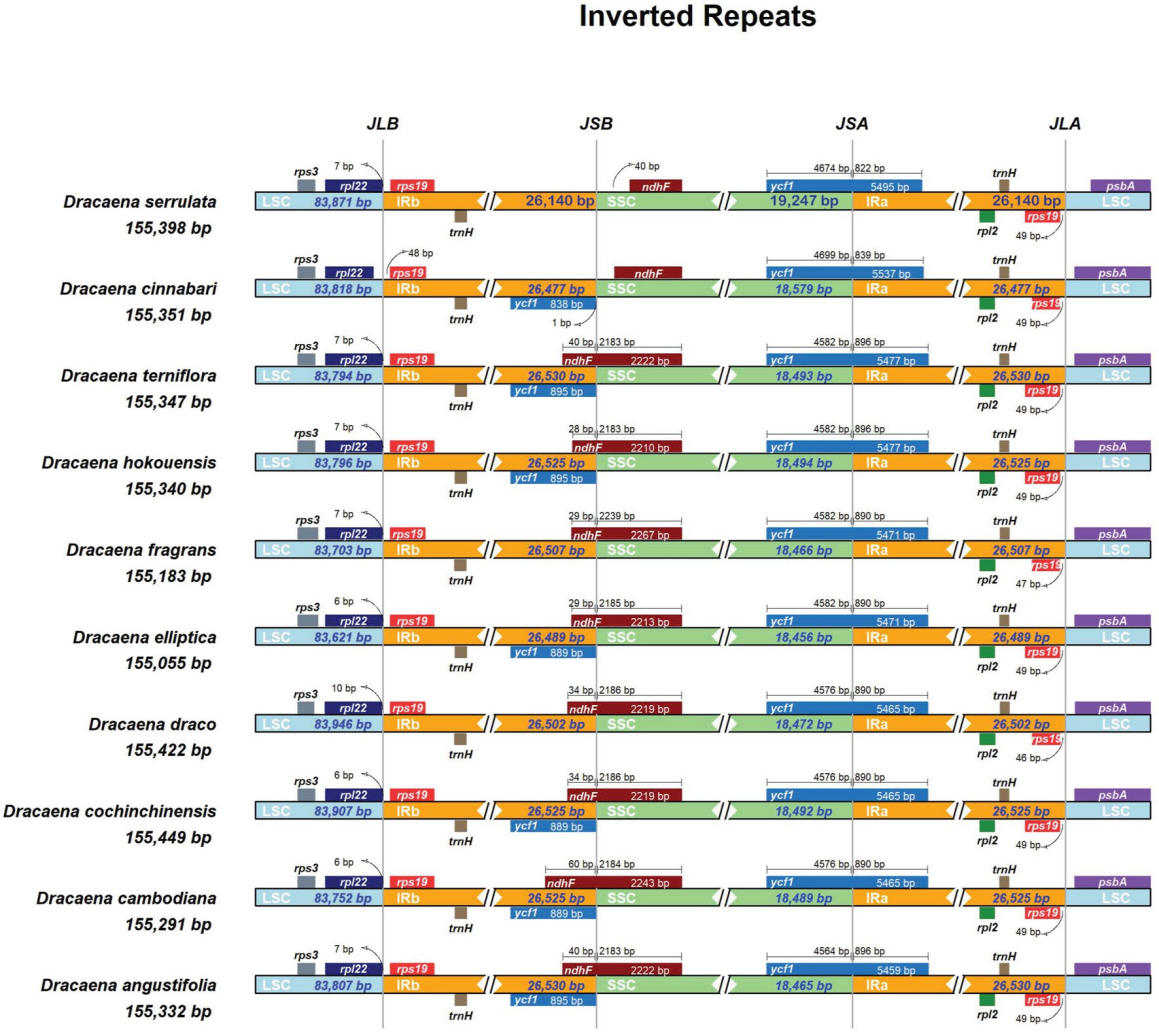
Distances between adjacent genes and junctions of the small single-copy (SSC), large single-copy (LSC), and two inverted repeats (IR) regions among *D. serrulata, D. cinnabari,* and related *Dracaena* species cp genomes. Boxes above and below the primary line indicate the adjacent border genes. The figure is not scaled regarding sequence length and only shows relative changes at or near the IR/SC borders.

Inverted repeat regions are considered the most conserved regions. The size variations in cp genomes occur due to expansion/contraction of IRs and single copy regions^76,90,91^. The four junctions (JLA, JLB, JSA, and JSB) between the single copy regions (LSC, SSC) and IRs (IRA, IRB) in cp genomes of *D. serrulata, D. cinnabari,* and others were comprehensively assessed. The IRs regions are remarkably conserved across all the cp genomes in the current study. The IRs regions' lengths correlate across all the compared cp genomes with only slight expansion and contraction (Fig. 8). The cp genomes of *D. serrulata* and *D. cinnabari* have the shortest IRs regions of 26,140 bp and 26,477 bp, respectively. In comparison, *D. angustifolia* and *D. terniflora* possess the most extended IRs regions of 26,530 bp. The positions of *rpl22* and *rps19* genes at JLB are similar in all the cp genomes with only one base (*D. elliptica*, *D. cochinchinensis*) and three bases (*D. draco*) differences. Interestingly, the *ndhF* gene was found 40 and 22 base pairs away from the JSB in SSC in cp genome of *D. serrulata* and *D. cinnabari* (Fig. 8)*.* In contrast, in other compared cp genomes it is found extended into IRb regions and overlaps with *ycf1* (28–80 bp) as found previously^92^. Similarly, the *ycf1* and *rpl22* genes at JSA and JSB are slightly variable across some cp genomes. Previous reports support the results^92–94^.

### Phylogenetic analysis

**Figure 9:**
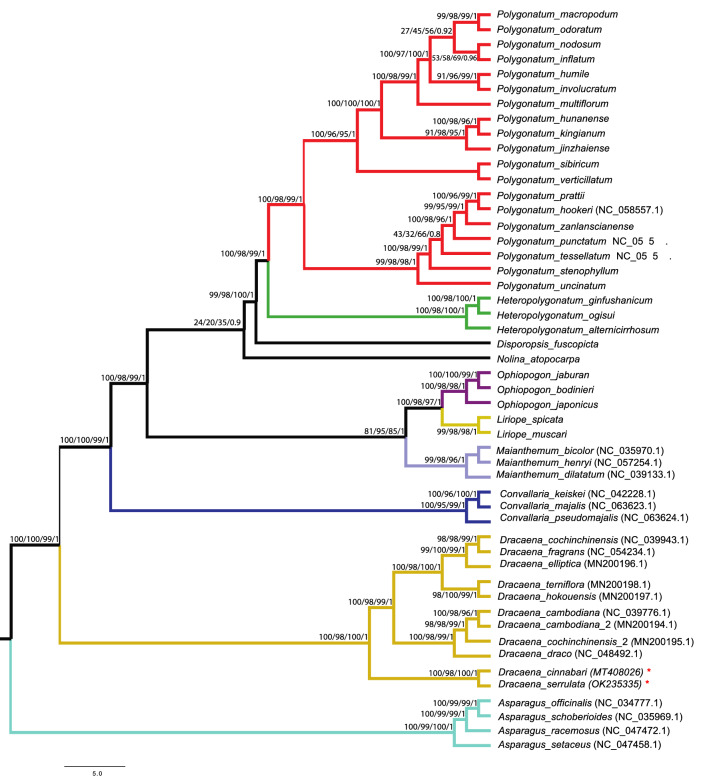
The phylogenetic tree is based on 46 complete cp genomes from subfamily Nolinoideae and four complete cp genomes from subfamily Asparagoidea as outgroups using neighbor-joining (NJ), maximum likelihood (ML), Bayesian inference (BI) and maximum parsimony (MP) methods.Numbers above the branches represent bootstrap values in NJ, ML, BI and MP trees, respectively. Different colors represent the subfamilies in Asparagaceae family.

Since the eighteenth century, the phylogenetic relationships among the *Dracaena* species have not been completely clarified and are still unclear. In Dracaena, significant morphological variation has been shown, with species generally^3^. Until recently, limited number of genetic markers such as chloroplast genes (*matK, rbcL* and intergenic spacer regions such as *rpl32*-*trnL*, *trnQ*-*rps16*, *psbA*-*trnH*, *trnL*-*trnF* etc.) were used to infer the phylogenetic relationships between the various *Dracaena* species such as *D*. *serrulata*, *D*. *cinnabari* and *D*. *draco,* etc. Therefore, additional genetic markers are required to determine the phylogenetic position of *D. cinnabari* and *D. serrulata*. Cp genomes as a super-barcode and concatenated protein coding genes with sufficient informative sites have been proven effective in resolving complicated phylogenetic relationships in various complex plant species^19^. Therefore, this study determined the phylogenetic dispositions of D. serrulata and D. cinnabari within the subfamily Nolinoideae by analyzing 46 complete cp genomes from subfamily Nolinoideae and four complete cp genomes from subfamily Asparagoidea as outgroups (Fig. 9) and 66 shared protein-coding genes (Fig. S1). Phylogenetic analysis using ML, BI, NJ and MP methods was performed. The phylogenetic analysis of complete genomes and shared protein coding genes revealed almost the same phylogenetic signals. In these phylogenetic trees (Figs. 9, S1), *D. serrulata* and *D. cinnabari* formed a single clade with high bootstrap value and BI support.

Moreover, the tree topology enabled inference of the relationship based on the phylogenetic studies conducted by Durán et al.^19^. The position of both *D. serrulata* and *D. cinnabari* confirms the previously published phylogeny described by Durán et al.^19^ that *D. serrulata* is more closely related to *D. cinnabari* than *D. draco* (Fig. 9). Furthermore, both trees revealed that *D. draco* is more closely related to *D. cochinchinensis* and *D. cambodiana*. Similar results were reported previously by Durán et al.^19^on the basis of chloroplast barcode genes such as *rbcL* and *matK* genes and intergenic spacer regions(*trnQ-rps16* and *rpl32-trnL*). However, another study by Lu and Morden^18^ based on combined chloroplast intergenic spacer regions using Bayesian analysis showed contradictory results to our study, where *D. serrulata* was closely related to *D. draco*. Furthermore, The earlier finding of Wang et al.^95^, who placed Liriope and Ophiopogon in the tribe Ophiopogoneae, is also supported by our phylogenetic trees Lun-Kai et al.^96^, Song‐Yun and Lun‐Kai^97^, and^98^ proposed that Ophiopogon and Liriope were closely related based on characteristics of the leaf epidermis and pollen as well as chromosomal counts. Even though our findings, based on the available cp genomes, clarified the phylogenetic relationships of some *D. serrulata* and *D. cinnabari*, more complete cp genome sequences are needed to resolve the comprehensive phylogenies of this genus because limited taxon sampling may produce discrepancies in tree topologies as reported earlier^99^.

## Conclusion

In the current study, the complete chloroplast genomes of *D. serrulata* and *D. cinnabari* were sequenced and elucidated for the first time. The overall gene order and cp genome organization were similar to nine *Dracaena* species. Repetitive sequences and SSRs were identified from the sequenced data and nine related cp genomes. In contrast, the highest number of repeats and SSRs were identified in *D. terniflora* and *D. serrulata*. Moreover, divergence is detected in intergenic spaces greater than in protein-coding regions of these cp genomes. Current results showed that the *D. serrulata* and *D. cinnabari* form a single clade. The whole cp genome sequencing of *D. serrulata* and *D. cinnabari* gives exciting insights and valuable data that may facilitate the identification of related species and answer taxonomic questions.

## Supplementary Information

Supplementary Information 1.

Supplementary Information 2.

## Data Availability

All data generated or analyzed during this study are included in this published article. The *D. serrulata* and *D. cinnabari* cp genomes were submitted to NCBI with accession numbers MT408026 and OK235335 respectively.
