# Supplementary material for: Complete chloroplast genome sequencing and comparative analysis of threatened dragon trees Dracaena serrulata and Dracaena cinnabari
Source: Sci Rep. 2022 Oct 6;12:16787. doi: 10.1038/s41598-022-20304-6 (PMC9537188; doi:10.1038/s41598-022-20304-6)
Supplement: Supplementary file 1 — Supplementary Information 1. [file 41598_2022_20304_MOESM1_ESM.docx]

**Figure S1.** Complete cp genome-based phylogenetic tree of *D. serrulata* and *D. cinnabari*. The entire genome dataset was analyzed using the maximum likelihood (ML) method. Numbers above the branches represent bootstrap values in the ML trees. Different colors represent subfamilies in Asparagaceae family.
