# Supplementary material for: Complete chloroplast genome sequencing and comparative analysis of threatened dragon trees Dracaena serrulata and Dracaena cinnabari
Source: Sci Rep. 2022 Oct 6;12:16787. doi: 10.1038/s41598-022-20304-6 (PMC9537188; doi:10.1038/s41598-022-20304-6)
Supplement: Supplementary file 2 — Supplementary Information 2. [file 41598_2022_20304_MOESM2_ESM.docx]

**Table S1**. Codon usage in *D. serrulata and D. cinnabari* cp genomes.

| Codon | | Amino acid | | Frequency | | Number | |
| --- | --- | --- | --- | --- | --- | --- | --- |
| DS | **DC** | **DS** | **DC** | **DS** | **DC** | **DS** | **DC** |
| GCA | GCA | A | A | 14.377 | 14.377 | 705 | 705 |
| GCC | GCC | A | A | 7.362 | 7.362 | 361 | 361 |
| GCG | GCG | A | A | 5.241 | 5.241 | 257 | 257 |
| GCT | GCT | A | A | 21.23 | 21.23 | 1041 | 1041 |
| TGC | TGC | C | C | 4.466 | 4.466 | 219 | 219 |
| TGT | TGT | C | C | 9.769 | 9.769 | 479 | 479 |
| GAC | GAC | D | D | 8.382 | 8.382 | 411 | 411 |
| GAT | GAT | D | D | 30.509 | 30.509 | 1496 | 1496 |
| GAA | GAA | E | E | 36.199 | 36.199 | 1775 | 1775 |
| GAG | GAG | E | E | 14.276 | 14.276 | 700 | 700 |
| TTC | TTC | F | F | 23.412 | 23.412 | 1148 | 1148 |
| TTT | TTT | F | F | 36.79 | 36.79 | 1804 | 1804 |
| GGA | GGA | G | G | 26.695 | 26.695 | 1309 | 1309 |
| GGC | GGC | G | G | 6.587 | 6.587 | 323 | 323 |
| GGG | GGG | G | G | 11.808 | 11.808 | 579 | 579 |
| GGT | GGT | G | G | 20.128 | 20.128 | 987 | 987 |
| CAC | CAC | H | H | 6.2 | 6.2 | 304 | 304 |
| CAT | CAT | H | H | 19.231 | 19.231 | 943 | 943 |
| ATA | ATA | I | I | 25.859 | 25.859 | 1268 | 1268 |
| ATC | ATC | I | I | 19.17 | 19.17 | 940 | 940 |
| ATT | ATT | I | I | 40.135 | 40.135 | 1968 | 1968 |
| AAA | AAA | K | K | 41.521 | 41.521 | 2036 | 2036 |
| AAG | AAG | K | K | 16.662 | 16.662 | 817 | 817 |
| CTA | CTA | L | L | 13.032 | 13.032 | 639 | 639 |
| CTC | CTC | L | L | 7.546 | 7.546 | 370 | 370 |
| CTG | CTG | L | L | 6.913 | 6.913 | 339 | 339 |
| CTT | CTT | L | L | 21.311 | 21.311 | 1045 | 1045 |
| TTA | TTA | L | L | 30.019 | 30.019 | 1472 | 1472 |
| TTG | TTG | L | L | 21.597 | 21.597 | 1059 | 1059 |
| ATG | ATG | M | M | 22.555 | 22.555 | 1106 | 1106 |
| AAC | AAC | N | N | 12.318 | 12.318 | 604 | 604 |
| AAT | AAT | N | N | 36.301 | 36.301 | 1780 | 1780 |
| CCA | CCA | P | P | 11.441 | 11.441 | 561 | 561 |
| CCC | CCC | P | P | 8.3 | 8.3 | 407 | 407 |
| CCG | CCG | P | P | 5.384 | 5.384 | 264 | 264 |
| CCT | CCT | P | P | 13.97 | 13.97 | 685 | 685 |
| CAA | CAA | Q | Q | 26.287 | 26.287 | 1289 | 1289 |
| CAG | CAG | Q | Q | 9.809 | 9.809 | 481 | 481 |
| AGA | AGA | R | R | 21.944 | 21.944 | 1076 | 1076 |
| AGG | AGG | R | R | 7.913 | 7.913 | 388 | 388 |
| CGA | CGA | R | R | 12.991 | 12.991 | 637 | 637 |
| CGC | CGC | R | R | 3.385 | 3.385 | 166 | 166 |
| CGG | CGG | R | R | 5.282 | 5.282 | 259 | 259 |
| CGT | CGT | R | R | 12.583 | 12.583 | 617 | 617 |
| AGC | AGC | S | S | 5.18 | 5.18 | 254 | 254 |
| AGT | AGT | S | S | 14.847 | 14.847 | 728 | 728 |
| TCA | TCA | S | S | 17.11 | 17.11 | 839 | 839 |
| TCC | TCC | S | S | 13.623 | 13.623 | 668 | 668 |
| TCG | TCG | S | S | 7.852 | 7.852 | 385 | 385 |
| TCT | TCT | S | S | 21.617 | 21.617 | 1060 | 1060 |
| ACA | ACA | T | T | 14.867 | 14.867 | 729 | 729 |
| ACC | ACC | T | T | 8.851 | 8.851 | 434 | 434 |
| ACG | ACG | T | T | 6.077 | 6.077 | 298 | 298 |
| ACT | ACT | T | T | 19.231 | 19.231 | 943 | 943 |
| GTA | GTA | V | V | 18.008 | 18.008 | 883 | 883 |
| GTC | GTC | V | V | 6.811 | 6.811 | 334 | 334 |
| GTG | GTG | V | V | 6.893 | 6.893 | 338 | 338 |
| GTT | GTT | V | V | 18.803 | 18.803 | 922 | 922 |
| TGG | TGG | W | W | 18.375 | 18.375 | 901 | 901 |
| TAC | TAC | Y | Y | 8.443 | 8.443 | 414 | 414 |
| TAT | TAT | Y | Y | 28.796 | 28.796 | 1412 | 1412 |
| TAA | TAA | * | * | 2.875 | 2.875 | 141 | 141 |
| TAG | TAG | * | * | 2.427 | 2.427 | 119 | 119 |
| TGA | TGA | * | * | 2.427 | 2.427 | 119 | 119 |

DS: *Dracaena serrulata,* DC*: Dracaena cinnabari*

**Table S2.** Average pairwise distance of complete chloroplast sequences from *D. cinnabari* with related species.

| Cp genomes | Average pairwise distance |
| --- | --- |
| Dracaena cinnabari |  |
| Dracaena serrulata | 0.0030340 |
| Dracaena cochinchinensis | 0.0077460 |
| Dracaena angustifolia | 0.0072960 |
| Dracaena cambodiana | 0.0070725 |
| Dracaena cochinchinensis | 0.0072426 |
| Dracaena elliptica | 0.0071493 |
| Dracaena hokouensis | 0.0074113 |
| Dracaena terniflora | 0.0072230 |
| Dracaena draco | 0.0075769 |
| Dracaena fragrans | 0.0077198 |

**Table S3.** Average pairwise distance of chloroplast shared genes from *D. cinnabari* with related species.

| Gene | *D. cinnabari* | *D. serrulata* | *D. cochinchinensis* | *D. angustifolia* | *D. cambodiana* | *D. cochinchinensis* | *D. elliptica* | *D. hokouensis* | *D. terniflora* | *D. draco* | *D. fragrans* |
| --- | --- | --- | --- | --- | --- | --- | --- | --- | --- | --- | --- |
| *accD* |  | 0.0067175075 | 0.0059096491 | 0.0141895141 | 0.0119824942 | 0.0119824942 | 0.0134329403 | 0.0059120045 | 0.0141895141 | 0.0218505137 | 0.0059096491 |
| *atpA* |  | 0.0000000000 | 0.0046130181 | 0.0052740007 | 0.0065974863 | 0.0072599963 | 0.0065974863 | 0.0065974863 | 0.0052740007 | 0.0072632368 | 0.0046130181 |
| *atpB* |  | 0.0000000000 | 0.0047282026 | 0.0047247639 | 0.0047247639 | 0.0047272947 | 0.0033712778 | 0.0081198335 | 0.0060805240 | 0.0054028375 | 0.0047282026 |
| *atpE* |  | 0.0000000000 | 0.0125046781 | 0.0024751834 | 0.0049632299 | 0.0049632299 | 0.0074500152 | 0.0074500152 | 0.0024751834 | 0.0049632299 | 0.0125046781 |
| *atpF* |  | 0.0021967570 | 0.0080503980 | 0.0043792502 | 0.0043772277 | 0.0058396428 | 0.0043772277 | 0.0058416827 | 0.0051105874 | 0.0051105874 | 0.0080503980 |
| *atpH* |  | 0.0000000000 | 0.0000000000 | 0.0000000000 | 0.0000000000 | 0.0000000000 | 0.0040827102 | 0.0040821149 | 0.0000000000 | 0.0000000000 | 0.0000000000 |
| *atpI* |  | 0.0000000000 | 0.0040454662 | 0.0040520044 | 0.0026952977 | 0.0026952977 | 0.0054036155 | 0.0040501855 | 0.0026952977 | 0.0040454662 | 0.0040454662 |
| *ccsA* |  | 0.0000000000 | 0.0026008990 | 0.0034721740 | 0.0025986876 | 0.0043419119 | 0.0034710373 | 0.0026008990 | 0.0026008990 | 0.0026008990 | 0.0026008990 |
| *cemA* |  | 0.0000000000 | 0.0062141125 | 0.0082942940 | 0.0020621902 | 0.0020621902 | 0.0062141125 | 0.0062141125 | 0.0062141125 | 0.0083136740 | 0.0062141125 |
| *matK* |  | 0.0092874138 | 0.0047246853 | 0.0036993684 | 0.0033578559 | 0.0036948138 | 0.0043729519 | 0.0040381233 | 0.0047166164 | 0.0036965262 | 0.0047587352 |
| *ndhA* |  | 0.0000000000 | 0.0078574656 | 0.0088332505 | 0.0069541368 | 0.0060197053 | 0.0069501928 | 0.0097611946 | 0.0088548422 | 0.0078856454 | 0.0078574656 |
| *ndhB* |  | 0.0000000000 | 0.0008972676 | 0.0008972676 | 0.0004483324 | 0.0004483324 | 0.0008979434 | 0.0008972676 | 0.0013468066 | 0.0004483324 | 0.0008972676 |
| *ndhC* |  | 0.0000000000 | 0.0027712038 | 0.0027712038 | 0.0027712038 | 0.0027712038 | 0.0027712038 | 0.0027712038 | 0.0027712038 | 0.0027712038 | 0.0027712038 |
| *ndhD* |  | 0.0072972291 | 0.0060143349 | 0.0039535980 | 0.0046138544 | 0.0046138544 | 0.0052791515 | 0.0039549871 | 0.0026337479 | 0.0059348269 | 0.0059428445 |
| *ndhE* |  | 0.0000000000 | 0.0032767661 | 0.0065788106 | 0.0065614505 | 0.0032767661 | 0.0065788106 | 0.0131895799 | 0.0065788106 | 0.0032767661 | 0.0032767661 |
| *ndhF* |  | 0.0000000000 | 0.0104926022 | 0.0081919910 | 0.0081953018 | 0.0095663414 | 0.0081996814 | 0.0095796747 | 0.0091117492 | 0.0081843331 | 0.0104926022 |
| *ndhG* |  | 0.0000000000 | 0.0094849518 | 0.0018862040 | 0.0037820723 | 0.0056778586 | 0.0018862040 | 0.0018862040 | 0.0018862040 | 0.0018862040 | 0.0094849518 |
| *ndhH* |  | 0.0000000000 | 0.0059552049 | 0.0025458044 | 0.0042501479 | 0.0051023196 | 0.0033958307 | 0.0025458044 | 0.0033986900 | 0.0042507810 | 0.0059552049 |
| *ndhI* |  | 0.0000000000 | 0.0074233036 | 0.0055499520 | 0.0036966934 | 0.0036966934 | 0.0055625380 | 0.0055499520 | 0.0055499520 | 0.0055625380 | 0.0074233036 |
| *ndhJ* |  | 0.0000000000 | 0.0042122546 | 0.0042122546 | 0.0000000000 | 0.0000000000 | 0.0042122546 | 0.0063234906 | 0.0042122546 | 0.0020981241 | 0.0042122546 |
| *ndhK* |  | 0.0000000000 | 0.0055741713 | 0.0055844700 | 0.0027870132 | 0.0027870132 | 0.0055741713 | 0.0041832828 | 0.0069942887 | 0.0027849407 | 0.0055741713 |
| *petB* |  | 0.0000000000 | 0.0068421228 | 0.0086441915 | 0.0064717597 | 0.0050270373 | 0.0050209444 | 0.0064674050 | 0.0079142053 | 0.0045532810 | 0.0068421228 |
| *petD* |  | 0.0000000000 | 0.0068260983 | 0.0062579653 | 0.0056858061 | 0.0062579653 | 0.0068261338 | 0.0062579978 | 0.0056858356 | 0.0056858061 | 0.0068260983 |
| *petG* |  | 0.0000000000 | 0.0000000000 | 0.0000000000 | 0.0000000000 | 0.0000000000 | 0.0000000000 | 0.0000000000 | 0.0000000000 | 0.0000000000 | 0.0000000000 |
| *petL* |  | 0.0000000000 | 0.0106864376 | 0.0106864376 | 0.0106864376 | 0.0106864376 | 0.0106864376 | 0.0106864376 | 0.0106864376 | 0.0106864376 | 0.0106864376 |
| *petN* |  | 0.0000000000 | 0.0114152674 | 0.0114152674 | 0.0114152674 | 0.0114152674 | 0.0114152674 | 0.0114152674 | 0.0114152674 | 0.0114152674 | 0.0114152674 |
| *psaA* |  | 0.0017797465 | 0.0022226092 | 0.0022228744 | 0.0013330928 | 0.0013330928 | 0.0031138713 | 0.0026683998 | 0.0026691224 | 0.0013330928 | 0.0022226092 |
| *psaB* |  | 0.0000000000 | 0.0022730178 | 0.0022730178 | 0.0022727320 | 0.0018175477 | 0.0018177762 | 0.0027289227 | 0.0022730178 | 0.0022727320 | 0.0022730178 |
| *psaC* |  | 0.0000000000 | 0.0165891302 | 0.0165891302 | 0.0082263341 | 0.0082263341 | 0.0082263341 | 0.0082263341 | 0.0082263341 | 0.0082263341 | 0.0082263341 |
| *psaI* |  | 0.0000000000 | 0.0000000000 | 0.0000000000 | 0.0000000000 | 0.0000000000 | 0.0000000000 | 0.0000000000 | 0.0000000000 | 0.0000000000 | 0.0000000000 |
| *psaJ* |  | 0.0000000000 | 0.0000000000 | 0.0000000000 | 0.0158567855 | 0.0158567855 | 0.0000000000 | 0.0017846544 | 0.0000000000 | 0.0017846544 | 0.0000000000 |
| *psbA* |  | 0.0000000000 | 0.0018858119 | 0.0018854051 | 0.0037764266 | 0.0037764266 | 0.0018858119 | 0.0028306019 | 0.0018854051 | 0.0037745699 | 0.0018858119 |
| *psbB* |  | 0.0000000000 | 0.0026250161 | 0.0032820533 | 0.0006550081 | 0.0006550081 | 0.0026257234 | 0.0039394755 | 0.0026249684 | 0.0006550081 | 0.0026250161 |
| *psbC* |  | 0.0000000000 | 0.0014081373 | 0.0014076182 | 0.0000000000 | 0.0000000000 | 0.0007036339 | 0.0007033842 | 0.0007033842 | 0.0000000000 | 0.0014081373 |
| *psbD* |  | 0.0000000000 | 0.0047235650 | 0.0037757196 | 0.0018855831 | 0.0028308037 | 0.0047235650 | 0.0047235650 | 0.0037757196 | 0.0037774190 | 0.0047235650 |
| *psbE* |  | 0.0000000000 | 0.0039761779 | 0.0080063390 | 0.0000000000 | 0.0000000000 | 0.0039761779 | 0.0080063390 | 0.0080063390 | 0.0000000000 | 0.0039761779 |
| *psbF* |  | 0.0000000000 | 0.0083956117 | 0.0083956117 | 0.0083956117 | 0.0083956117 | 0.0169184377 | 0.0083956117 | 0.0083956117 | 0.0171211882 | 0.0083956117 |
| *psbH* |  | 0.0000000000 | 0.0090879864 | 0.0090992675 | 0.0045242800 | 0.0045242800 | 0.0045242800 | 0.0090992675 | 0.0090992675 | 0.0045242800 | 0.0090879864 |
| *psbI* |  | 0.0000000000 | 0.0000000000 | 0.0000000000 | 0.0000000000 | 0.0000000000 | 0.0000000000 | 0.0000000000 | 0.0000000000 | 0.0000000000 | 0.0000000000 |
| *psbJ* |  | 0.0000000000 | 0.0000000000 | 0.0000000000 | 0.0081673262 | 0.0000000000 | 0.0000000000 | 0.0000000000 | 0.0000000000 | 0.0000000000 | 0.0000000000 |
| *psbK* |  | 0.0000000000 | 0.0054581302 | 0.0000000000 | 0.0000000000 | 0.0000000000 | 0.0000000000 | 0.0000000000 | 0.0000000000 | 0.0000000000 | 0.0054581302 |
| *psbL* |  | 0.0000000000 | 0.0000000000 | 0.0000000000 | 0.0000000000 | 0.0000000000 | 0.0000000000 | 0.0000000000 | 0.0087026469 | 0.0000000000 | 0.0000000000 |
| *psbM* |  | 0.0000000000 | 0.0000000000 | 0.0000000000 | 0.0000000000 | 0.0000000000 | 0.0000000000 | 0.0000000000 | 0.0000000000 | 0.0000000000 | 0.0000000000 |
| *psbN* |  | 0.0000000000 | 0.0000000000 | 0.0000000000 | 0.0000000000 | 0.0000000000 | 0.0000000000 | 0.0000000000 | 0.0000000000 | 0.0000000000 | 0.0000000000 |
| *psbT* |  | 0.0000000000 | 0.0000000000 | 0.0000000000 | 0.0000000000 | 0.0000000000 | 0.0000000000 | 0.0000000000 | 0.0000000000 | 0.0000000000 | 0.0000000000 |
| *psbZ* |  | 0.0000000000 | 0.0000000000 | 0.0000000000 | 0.0000000000 | 0.0000000000 | 0.0000000000 | 0.0000000000 | 0.0000000000 | 0.0000000000 | 0.0000000000 |
| *rbcL* |  | 0.0000000000 | 0.0104960558 | 0.0083797751 | 0.0041775940 | 0.0048769065 | 0.0090939883 | 0.0083797751 | 0.0097901660 | 0.0034805726 | 0.0104960558 |
| *rpl2* |  | 0.0000000000 | 0.0013584893 | 0.0013584893 | 0.0013584893 | 0.0013584893 | 0.0013584893 | 0.0027188901 | 0.0020392818 | 0.0020384505 | 0.0013584893 |
| *rpl14* |  | 0.0000000000 | 0.0000000000 | 0.0027175917 | 0.0000000000 | 0.0000000000 | 0.0027261818 | 0.0027175917 | 0.0027175917 | 0.0000000000 | 0.0000000000 |
| *rpl16* |  | 0.0049251993 | 0.0198516164 | 0.0183952013 | 0.0296506846 | 0.0253429448 | 0.0205412026 | 0.0200681584 | 0.0197523520 | 0.0212122261 | 0.0198516164 |
| *rpl20* |  | 0.0000000000 | 0.0085648338 | 0.0056853664 | 0.0056853664 | 0.0056853664 | 0.0056853664 | 0.0056853664 | 0.0085397294 | 0.0057064194 | 0.0085648338 |
| *rpl22* |  | 0.0000000000 | 0.0196979146 | 0.0130440187 | 0.0097736751 | 0.0097711710 | 0.0097711710 | 0.0196416825 | 0.0230150916 | 0.0130659370 | 0.0196979146 |
| *rpl23* |  | 0.0000000000 | 0.0035550007 | 0.0035550007 | 0.0035550007 | 0.0107138967 | 0.0035550007 | 0.0035550007 | 0.0035550007 | 0.0035550007 | 0.0035550007 |
| *rpl32* |  | 0.0000000000 | 0.0057746285 | 0.0000000000 | 0.0000000000 | 0.0000000000 | 0.0057746285 | 0.0057812983 | 0.0058122507 | 0.0116166477 | 0.0057746285 |
| *rpl33* |  | 0.0000000000 | 0.0097581617 | 0.0097581617 | 0.0097581617 | 0.0147104446 | 0.0197767882 | 0.0097581617 | 0.0097581617 | 0.0147578545 | 0.0097581617 |
| *rpl36* |  | 0.0000000000 | 0.0000000000 | 0.0088642734 | 0.0088642734 | 0.0088642734 | 0.0000000000 | 0.0088642734 | 0.0177719805 | 0.0000000000 | 0.0000000000 |
| *rpoA* |  | 0.0000000000 | 0.0079905548 | 0.0079905548 | 0.0059789590 | 0.0079863357 | 0.0049785002 | 0.0079837905 | 0.0089936890 | 0.0079888826 | 0.0079905548 |
| *rpoB* |  | 0.0000000000 | 0.0046723020 | 0.0024885970 | 0.0021761247 | 0.0015537785 | 0.0024874558 | 0.0037365680 | 0.0021766097 | 0.0027989370 | 0.0046723020 |
| *rpoC1* |  | 0.0000000000 | 0.0072435062 | 0.0057850754 | 0.0065200184 | 0.0072486998 | 0.0061500375 | 0.0050619245 | 0.0057860448 | 0.0050621390 | 0.0072435062 |
| *rpoC2* |  | 0.0060758759 | 0.0082357170 | 0.0087213698 | 0.0060490190 | 0.0062914118 | 0.0072621173 | 0.0079947037 | 0.0079916577 | 0.0062914118 | 0.0082357170 |
| *rps2* |  | 0.0000000000 | 0.0028192600 | 0.0000000000 | 0.0014087000 | 0.0014087000 | 0.0056470379 | 0.0014077463 | 0.0000000000 | 0.0000000000 | 0.0028192600 |
| *rps3* |  | 0.0000000000 | 0.0076551526 | 0.0045830393 | 0.0061174113 | 0.0076519665 | 0.0076551526 | 0.0045830393 | 0.0045830393 | 0.0091962774 | 0.0076551526 |
| *rps4* |  | 0.0000000000 | 0.0033149706 | 0.0033149706 | 0.0016538268 | 0.0016538268 | 0.0033149706 | 0.0033149706 | 0.0049961808 | 0.0016538268 | 0.0033149706 |
| *rps7* |  | 0.0000000000 | 0.0000000000 | 0.0000000000 | 0.0000000000 | 0.0000000000 | 0.0021421955 | 0.0000000000 | 0.0021411194 | 0.0000000000 | 0.0000000000 |
| *rps8* |  | 0.0000000000 | 0.0000000000 | 0.0000000000 | 0.0050560083 | 0.0025124267 | 0.0025124267 | 0.0000000000 | 0.0000000000 | 0.0025124267 | 0.0000000000 |
| *rps11* |  | 0.0000000000 | 0.0072298315 | 0.0072298315 | 0.0048250229 | 0.0096943844 | 0.0072460065 | 0.0072460065 | 0.0048142737 | 0.0048207332 | 0.0072298315 |
| *rps12* |  | 0.0000000000 | 0.0000000000 | 0.0010964431 | 0.0000000000 | 0.0000000000 | 0.0000000000 | 0.0010964431 | 0.0010964431 | 0.0000000000 | 0.0000000000 |
| *rps14* |  | 0.0000000000 | 0.0000000000 | 0.0033060604 | 0.0000000000 | 0.0000000000 | 0.0000000000 | 0.0000000000 | 0.0000000000 | 0.0000000000 | 0.0000000000 |
| *rps15* |  | 0.0000000000 | 0.0149593332 | 0.0149593332 | 0.0149725782 | 0.0149725782 | 0.0111901437 | 0.0149593332 | 0.0187484306 | 0.0149484450 | 0.0149593332 |
| *rps16* |  | 0.0026863944 | 0.0126497456 | 0.0144544090 | 0.0108219228 | 0.0117190969 | 0.0126611842 | 0.0117004762 | 0.0126313400 | 0.0117427466 | 0.0126497456 |
| *rps18* |  | 0.0000000000 | 0.0098984240 | 0.0000000000 | 0.0032839051 | 0.0000000000 | 0.0032873064 | 0.0000000000 | 0.0000000000 | 0.0032744977 | 0.0041254050 |
| *rps19* |  | 0.0179723864 | 0.0000000000 | 0.0000000000 | 0.0000000000 | 0.0000000000 | 0.0000000000 | 0.0000000000 | 0.0000000000 | 0.0000000000 | 0.0000000000 |
| *ycf1* |  | 0.0195625622 | 0.0223102715 | 0.0214728570 | 0.0207976211 | 0.0204934316 | 0.0225185317 | 0.0219836646 | 0.0216721629 | 0.0216193725 | 0.0223102715 |
| *ycf2* |  | 0.0000000000 | 0.0014835130 | 0.0010381318 | 0.0008897599 | 0.0007413452 | 0.0008923965 | 0.0008896182 | 0.0010379664 | 0.0008925391 | 0.0014835130 |
| *ycf3* |  | 0.0000000000 | 0.0093308306 | 0.0051893186 | 0.0048188510 | 0.0040830003 | 0.0089936809 | 0.0048183983 | 0.0051921539 | 0.0037112724 | 0.0093308306 |
| *ycf4* |  | 0.0000000000 | 0.0018030677 | 0.0054330989 | 0.0000000000 | 0.0000000000 | 0.0000000000 | 0.0018062099 | 0.0018062099 | 0.0000000000 | 0.0018030677 |

**Table S4.** Gene bank accession numbers of cp genomes included in the phylogenetic tree

| S. No | Name | Accession |
| --- | --- | --- |
| 1 | *Agave americana* | NC_032053.1 |
| 2 | *Agave attenuata* | NC_032696.1 |
| 3 | *Agave hybrid cultivar* | NC_045534.1 |
| 4 | *Agave virginica* | NC_032707.1 |
| 5 | *Anemarrhena asphodeloides* | NC_032698.1 |
| 6 | *Anthericum ramosum* | NC_035972.1 |
| 7 | *Aphyllanthes monspeliensis* | NC_035968.1 |
| 8 | *Asparagus filicinus* | NC_046783.1 |
| 9 | *Asparagus filicinus (reversed)* | NC_046783.1 |
| 10 | *Asparagus officinalis* | NC_034777.1 |
| 11 | *Asparagus racemosus* | NC_047472.1 |
| 12 | *Asparagus schoberioides* | NC_035969.1 |
| 13 | *Asparagus setaceus* | NC_047458.1 |
| 14 | *Beschorneria septentrionalis* | NC_032699.1 |
| 15 | *Camassia scilloides* | NC_032700.1 |
| 16 | *Chlorogalum pomeridianum* | NC_032701.1 |
| 17 | *Chlorophytum comosum* | NC_053844.1 |
| 18 | *Convallaria keiskei* | NC_042228.1 |
| 19 | *Cordyline indivisa* | NC_035998.1 |
| 20 | *Dracaena angustifolia* | MN200193.1 |
| 21 | *Dracaena cambodiana* | MN200194.1 |
| 22 | *Dracaena cinnabari* | OK235335 |
| 23 | *Dracaena cochinchinensis* | MF943127.1 |
| 24 | *Dracaena draco* | MN990038.1 |
| 25 | *Dracaena elliptica* | MN200196.1 |
| 26 | *Dracaena fragrans* | MW123093.1 |
| 27 | *Dracaena hokouensis* | MN200197.1 |
| 28 | *Dracaena serrulata* | MT408026 |
| 29 | *Dracaena terniflora* | MN200198.1 |
| 30 | *Eustrephus latifolius* | NC_025305.1 |
| 31 | *Hesperaloe campanulata* | NC_032702.1 |
| 32 | *Hesperaloe parviflora* | NC_032703.1 |
| 33 | *Hesperocallis undulata* | NC_032704.1 |
| 34 | *Hesperoyucca whipplei* | NC_032705.1 |
| 35 | *Hosta capitata* | NC_045519.1 |
| 36 | *Hosta clausa* | NC_046896.1 |
| 37 | *Hosta jonesii* | NC_046897.1 |
| 38 | *Hosta minor* | NC_035999.1 |
| 39 | *Hosta plantaginea* | NC_053555.1 |
| 40 | *Hosta ventricosa* | NC_032706.1 |
| 41 | *Hosta venusta* | NC_046895.1 |
| 42 | *Hosta yingeri* | NC_039976.1 |
| 43 | *Liriope muscari* | NC_045384.1 |
| 44 | *Liriope spicata* | NC_042227.1 |
| 45 | *Maianthemum bicolor* | NC_035970.1 |
| 46 | *Maianthemum dilatatum* | NC_039133.1 |
| 47 | *Milla biflora* | NC_036000.1 |
| 48 | *Nolina atopocarpa* | NC_032708.1 |
| 49 | *Ophiopogon bodinieri* | NC_051508.1 |
| 50 | *Ophiopogon jaburan* | NC_049870.1 |
| 51 | *Ophiopogon japonicus* | NC_049869.1 |
| 52 | *Polygonatum cirrhifolium* | NC_053687.1 |
| 53 | *Polygonatum cyrtonema* | NC_028429.1 |
| 54 | *Polygonatum kingianum* | NC_047406.1 |
| 55 | *Polygonatum odoratum* | NC_050926.1 |
| 56 | *Polygonatum sibiricum* | NC_029485.1 |
| 57 | *Polygonatum stenophyllum* | NC_035995.1 |
| 58 | *Polygonatum verticillatum* | NC_028523.1 |
| 59 | *Schoenolirion croceum* | NC_032710.1 |
| 60 | *Speirantha gardenii* | NC_053784.1 |
| 61 | *Yucca brevifolia* | NC_032711.1 |
| 62 | *Yucca filamentosa* | NC_032712.1 |
| 63 | *Yucca queretaroensis* | NC_032713.1 |
| 64 | *Yucca schidigera* | NC_032714.1 |
